# Perceived COVID-19 pandemic impact and protective factors predicting patient-reported depression and anxiety in individuals living with cancer

**DOI:** 10.1186/s41687-023-00571-1

**Published:** 2023-03-16

**Authors:** Erica E. Fortune, Alexandra K. Zaleta, Victoria G. Morris

**Affiliations:** Cancer Support Community, Research and Training Institute, 520 Walnut Street, Suite 1170, Philadelphia, PA 19106 USA

**Keywords:** Anxiety, Cancer, COVID-19, Depression, Health-related quality of life, Hope, Oncology, Patient-reported outcomes, PROMIS-29, Resilience

## Abstract

**Background:**

The COVID-19 pandemic presents a unique, amplified threat to those living with a cancer diagnosis, but personal factors may play a role in how this affects well-being. This cross-sectional study (1) describes the impacts of COVID-19 on cancer patients’ lives, and (2) explores the extent to which specific impacts of COVID-19 and noted protective factors, hope and resilience, predict two crucial patient-reported outcomes, depression and anxiety, after controlling for relevant sociodemographic and clinical factors.

**Methods:**

520 cancer patients and survivors in the U.S. completed an online survey during the first year of the pandemic and answered questions about COVID-19 areas of impact, psychological well-being, hope, and resilience. Hierarchical regression analyses were used to analyze the unique impact of each group of variables on patient-reported levels of depression and anxiety during the pandemic.

**Results:**

Participants strongly endorsed COVID-19 impact across several areas of life, especially social activity, well-being, and ability to acquire basic essentials. Regression models explained a substantial amount of variance in patient-reported depression (*R*^*2*^ = .50, *p* < *.*001) and anxiety (*R*^*2*^ = .44, *p* < *.*001), revealing COVID-19 financial impact as a significant predictor of depression (β = 0.07), and COVID-19 family impact as a significant predictor of anxiety (β = 0.14), even after controlling for the effects of relevant sociodemographic and clinical variables. Additionally, resilience and hope were the largest predictors of both depression (β = − 0.19 and − 0.37, respectively) and anxiety (β = − 0.18 and − 0.29), suggesting that they account for unique variance in patient-reported mental health during the COVID-19 pandemic and might serve as important protective factors.

**Conclusions:**

The current results add to existing literature documenting the significant effect of COVID-19 on those living with cancer. COVID-19 impact, including financial and family well-being, as well as positive psychological constructs, hope and resilience, play a crucial role in levels of patient-reported depression and anxiety during the pandemic. As COVID-19 continues to evolve, health care providers should routinely assess psychological well-being and needs related to COVID-19 financial and family impact in an effort to appropriately align individuals with resources and support, and consider how hope and resilience can be fostered to serve as psychological buffers during this time.

## Background

COVID-19 has substantially influenced almost all aspects of life, resulting in greater physical, financial, social, and psychological distress in the general population [1, 2]. Those living with a cancer diagnosis have been found to be more susceptible to severe cases of COVID-19 infection due to weakened immune systems, common comorbidities, and being of older age [3, 4]. The cancer community has also been forced to navigate abrupt and sometimes extreme modifications to their cancer care and treatment routines [5], resulting in greater concern and uncertainty regarding their future prognosis [6] as well as the potential for increased cancer-related mortality [7].

Prior to the COVID-19 pandemic, approximately 8–24% of cancer patients presented with clinical levels of depression [8] and 10% with anxiety [9]—rates that exceed that of the general population. Depression and anxiety have been recognized as core patient-reported outcomes associated with long-term well-being in those living with cancer [10, 11] and are even more essential to assess in the face of the pandemic. The depression and anxiety that cancer patients experience not only jeopardizes quality of life, but also creates a vulnerability to experiencing additional psychological distress in response to the pandemic, as past psychological health has been identified as a predictor of well-being during COVID-19 [1]. Moreover, while global mitigation strategies (e.g., lockdowns and social distancing) reduce the risk of COVID-19 infection, these strategies also act as a natural catalyst for isolation and related feelings of loneliness, which is a key area of concern for the cancer population in general [12, 13], and even more so during the pandemic [14, 15].

In the face of these risk factors for psychological distress during the pandemic, there are also notable protective factors to take into consideration, including individuals’ sense of hope and resilience. Having positive coping mechanisms [16] and higher levels of hope [17] have been shown to attenuate COVID-19 related distress and enhance well-being among the general population. Resilience has also been posited to act as a protective factor for cancer patients during the pandemic [18–20], and pre-pandemic literature demonstrates that resilience is associated with lower psychological distress in cancer patients [21], even for those experiencing advanced/metastatic disease [22, 23]. Data collected from the general population across multiple countries during the COVID-19 pandemic further suggests that resilience and hope are associated with better psychological well-being during this time [24].

The current study explores the relationship between COVID-19 impact and depression and anxiety in those living with cancer, accounting for established risk and protective factors for psychological well-being. Specifically, this study has three aims: (1) describe key areas of COVID-19 impact in the cancer population; (2) examine the extent to which key areas of COVID-19 impact predict patient-reported depression and anxiety, after controlling for the influence of known sociodemographic and clinical history risk factors; and (3) determine whether protective factors (i.e., resilience and hope) serve as unique predictors of patient-reported anxiety and depression, after controlling for sociodemographic and clinical risk factors as well as COVID-19 impacts.

We hypothesized that: (1) respondents will endorse multiple areas of COVID-19 impact; (2) greater COVID-19 impacts will be significantly associated with greater depression and anxiety; (3a) greater positive protective factors (hope and resilience) will be associated with lower depression and anxiety; and, (3b) hope and resilience will explain a significant amount of unique variance in patient-reported depression and anxiety, beyond the impact of sociodemographic, clinical, and COVID-19 impact factors.

## Methods

### Participants

520 cancer patients and survivors in the U.S. completed an online survey between September 2020 and December 2020 about their experiences during the COVID-19 pandemic. Participants were recruited through referrals from Cancer Support Community’s (CSC) U.S.-based network partners, including Cancer Support Community and Gilda’s Club partners, as well as CSC’s Cancer Experience Registry (an online, community-based research initiative examining the emotional, physical, practical, and financial impact of cancer), social media, and advocacy partnerships. Individuals ages 18 years and older who were ever diagnosed with cancer and who could read English were eligible to participate. These data are part of a longitudinal study with multiple waves of data collection; this study presents results from baseline data collection. Ethics approval was obtained from NORC at the University of Chicago Institutional Review Board (IRB00000967; Protocol #20.08.21). All procedures performed in studies involving human participants were in accordance with the ethical standards of the institutional research committee and with the 1964 Helsinki declaration and its later amendments or comparable ethical standards. Participants provided consent prior to beginning the survey.

### Measures

#### Sociodemographic background, clinical history, and COVID-19 status

Participants provided sociodemographic and clinical background information: age, gender identity, race, ethnicity, education, employment status, household income, self-reported cancer diagnosis, stage at diagnosis, current cancer status (experiencing cancer for the first time, experiencing cancer recurrence/relapse, currently in remission), years since diagnosis, and types of treatments ever received (past and current). Overall health status was assessed using a single item (In general, would you say your health is: 1 = *Poor*; 2 = *Fair*; 3 = *Good*; 4 = *Very Good*; 5 = *Excellent*). Participants indicated their COVID-19 testing and outcome status since the beginning of the pandemic by responding to two statements: (1) “I have been tested for COVID-19”; (2) “I tested positive for COVID-19.” Response options included: yes, no, I don’t know, prefer not to share.

#### COVID-19 impacts

Participants were asked the extent to which various aspects of their life (e.g., work, relationships with others, medical care, etc.) were impacted by the COVID pandemic, for 20 items that were constructed by the research team in consultation with a project advisory committee with expertise in oncology, psychology, and behavioral science. Participants rated impact using a 5-point scale (1 = *Very Negatively*; 5 = *Very Positively*) and could also indicate if an item was not applicable (e.g., “My parenting”).

#### Connor-Davidson Resilience Scale (CD-RISC-10^©^) [25]

Resilience was assessed using the 10-item version of the CD-RISC, which requires participants to indicate how true each statement is of their ability to handle hardships (0 = *Not true at all*; 4 = *True nearly all the time*). Higher scores (range: 0–40) reflect greater resilience and ability to handle hardships, with previous research showing that pre-pandemic U.S. adults in the general population average between 30.8 and 33.5 on the CD-RISC, while normative cancer patients in the U.S. receiving active treatment have a Mean = 32.2 [25].

#### Herth Hope Index (HHI) [26]

Participants rated 12 items (1 = *Strongly disagree*; 4 = *Strongly agree*) designed to measure hope in adults in clinical settings, with higher scores (range 12–48) indicating higher levels of hope. HHI scores have been documented in cancer patients pre-pandemic (*M* = 39.7) [27] as well as in multinational general populations (*M* = 38.7) during the pandemic [24].

#### Patient-reported outcomes measurement information system 29 profile V2.0 (PROMIS-29): depression and anxiety subscales [28]

Participants completed the 4-item Depression and 4-item Anxiety subscales of the PROMIS-29, indicating how often they experienced specific symptoms during the past 7 days (1 = *Never*; 5 = *Always*). Scale scores are converted to standardized T scores (Mean = 50, *SD* = 10; normative reference groups are the U.S. general population). Higher scores correspond to greater symptom burden, with roughly 15% of the general population reporting moderate to severe symptom burden (≥ 1SD above the Mean). Pre-pandemic levels of depression (M = 48.5) and anxiety (M = 49.2) have also been documented in the U.S. cancer population [29].

### Analysis

Data analysis was conducted using IBM SPSS Statistics 24.0. Descriptive statistics were calculated for study variables. To group the COVID-19 impact items for purposes of regression modeling, Exploratory Factor Analysis (EFA) using direct oblique rotation and principal axis factoring was conducted (see Results section). Then, composite scores for each COVID-19 impact factor were calculated by dichotomizing each item, such that the lowest response options (*Very Negatively* and *Somewhat Negatively*) were coded as 1 while all other values (*Not at All*, *Somewhat Positively*, *Very Positively*, *Not Applicable*) were coded as 0, and then summing together the items within each factor. Bivariate relationships between dependent variables (depression and anxiety) and hypothesized independent variables were investigated using Pearson correlations to substantiate inclusion in subsequent regression analyses. Correlations were considered large if *r* ≥ 0.50, medium if *r* = 0.30–0.49, and small if *r* = 0.10–0.29 [30]; all independent variables exhibiting at least a small effect size were entered as predictors into two hierarchical linear regressions, applying a block entry method. Casewise deletion was used for participants who did not have complete data. Model 1 assessed sociodemographic variables (Block 1), clinical history (Block 2), COVID-19 impacts (Block 3), and protective factors (Block 4) hypothesized to influence levels of patient-reported depression in the cancer population during the COVID-19 pandemic. Model 2 incorporated the same predictors as Model 1, with patient-reported anxiety as the outcome variable.

## Results

### Descriptive analyses

#### Participant sociodemographics, clinical history, and COVID-19 status

Participant descriptive statistics are presented in Table 1. Participants were predominantly women (76%), White (81%), with a bachelor’s degree or higher (59%). Average participant age was 60 years (SD = 12; range = 20–88), and average time since cancer diagnosis was 9.1 years (SD = 7.8; range =  < 1–56 years). The most commonly represented diagnoses included hematologic (29%) and breast (29%) cancers, with 29% of participants reporting active cancer (i.e., first time cancer or relapse). The majority of participants reported good (37%) or fair (30%) health overall. Almost half of participants (49%) had ever been tested for COVID-19, but only 3% reported ever testing positive.

**Table 1:** Descriptive characteristics of the sample (N = 520)

	Category/range	*M/n*	*SD*/*%*^a^
Age	Range 20–88	M = 60.0	SD = 12.1
Gender	Woman	393	76%
	Man	126	24%
	Genderqueer/non-binary	1	< 1%
Race	White	429	83%
	Black or African American	35	7%
	Asian or South Asian	16	3%
	American Indian or Alaskan Native (AIAN)	2	< 1%
	Other/Multiple races	27	5%
	Prefer not to share	11	2%
Ethnicity	Hispanic or Latino/a	30	6%
Education	No college	37	7%
	Some college	105	20%
	Associate or bachelor’s degree	200	39%
	Graduate or professional degree	173	33%
	Prefer not to share	5	1%
Annual income	< $20 K	39	8%
	$20–39 K	86	17%
	$40–59 K	76	15%
	$60–79 K	53	10%
	$80–99 K	51	10%
	$100 K +	123	24%
	Prefer not to share/Don’t know	92	18%
Employment	Full-time	123	24%
	Part-time	52	10%
	Retired	193	38%
	Disability	102	20%
	Unemployed	50	10%
Cancer diagnosis	Hematological Cancers	150	29%
	Breast Cancer	148	29%
	Gynecologic Cancers	36	7%
	Prostate Cancer	31	6%
	Lung Cancer	29	6%
	Other Cancer	29	6%
	Colorectal Cancer	24	5%
	Head and Neck Cancers	11	2%
	Melanoma	11	2%
	Thyroid Cancer	9	2%
	Brain Tumor/Central Nervous System	8	2%
	Skin Cancer	6	1%
	Other Cancers^b^	28	6%
Cancer status^c^	I am currently experiencing cancer for the first time	64	12%
	I am currently experiencing cancer recurrence/relapse	86	17%
	I am in remission or have no current evidence of disease	318	61%
	I don’t know/Other	50	10%
	Missing	2	< 1%
Years since diagnosis	Range 0–56	M = 9.1	SD = 7.8
	2 years or less	103	20%
	3–5 years	94	18%
	6–9 years	124	24%
	10–19 years	156	30%
	20 or more years	43	8%
Stage at diagnosis	0 (microscopic)	22	4%
	I (Small and removed by surgery)	88	17%
	II (extension to lymph nodes)	88	17%
	III (locally advanced)	112	22%
	IV (metastatic/widespread)	82	16%
	Other/Not Applicable	102	20%
	Don’t know	26	5%
Current stage^c^	Localized disease (has not spread)	48	9%
	Metastatic/Stage IV (has spread)	81	16%
	My cancer doesn’t have a stage/Not applicable	59	11%
	Don’t know	14	3%
Ever metastatic	No	384	74%
	Yes	124	24%
	Don’t know	12	2%
Treatment history	Current chemotherapy	60	12%
	Current radiation therapy	11	2%
	Current hormonal therapy	50	10%
	Current oral therapy	92	18%
	Current immunotherapy	46	9%
	Past chemotherapy	333	64%
	Past radiation therapy	259	50%
	Past hormonal therapy	91	18%
	Past oral therapy	75	14%
	Past immunotherapy	63	12%
	Past surgery	345	66%
General health	Range 0–5	M = 2.9	SD = 0.9
	Poor	21	4%
	Fair	157	30%
	Good	190	37%
	Very good	134	26%
	Excellent	18	4%

^a^Percentages might not add up to 100% due to rounding or missing data

^b^Other cancer diagnoses with < 1% prevalence included bladder, kidney, sarcoma, anal, gastrointestinal, pancreatic, among others

^c^Current status is dichotomized for regression analyses: active/current cancer (n = 150); in remission/other (n = 368); Current stage question not seen by those who indicated they are in remission or have no current evidence of disease. Percentages still reported out of total sample size (N = 520)

#### COVID-19 impacts

Participants strongly endorsed COVID-19 impact across several areas of life (Table 2). At an item level, the greatest areas of impact (*somewhat* or *very negatively* impacted by COVID-19) included the ability to engage in recreational activities (88%), feeling connected to others (76%), mental health (73%), friendships (63%), ability to secure basic essentials (56%), and medical care (56%). The least frequently endorsed items of COVID-19 impact included education or training (16%), housing (19%), and spirituality or religiosity (29%). The EFA of the items resulted in a 5-factor solution (TLI = 0.95, RMSR = 0.02, RMSEA = 0.05) explaining 51% of the item variance after dropping items with low endorsement and low factor loadings (< 0.30): Finances (e.g., work stability and paying bills), Family (e.g., relationship with family and ability to care for them), Well-Being (e.g., mental and physical health), Essential Needs (e.g., securing basic essentials and health care), and Social Activity (e.g., recreational activity and friendships). Note that the Well-Being factor was excluded from regression analyses due to redundancy with outcome variables.

**Table 2:** COVID-19 impact item endorsement and ranking by category

Items by category^a^	Overall Ranking^b^	% endorsement (*Very*—*Somewhat Negatively*)	N/A (%)
**Social activity**			
My ability to engage in recreational activities	1	87.1	1.0
How connected I feel to others	2	75.8	0.8
My friendships	4	63.3	1.0
**Well-being**			
My mental health	3	71.9	1.0
My physical health	7	52.7	1.5
My self-care	10	39.6	2.7
My ability to take care of household chores	14	28.7	2.3
**Essential needs**			
Ability to secure basic essentials	5	56.4	1.2
My medical care	6	55.8	1.0
My ability to obtain needed health care	9	50.8	1.0
**Family**			
My family relationships	8	52.5	2.1
My ability to care for my family	12	32.3	15.6
My relationship with my spouse or partner	17	24.8	27.3
My parenting	18	19.0	37.5
**Finances**			
My finances (e.g., ability to pay bills)	11	37.5	2.7
My work (e.g., loss of hours, loss of job, ability to obtain employment, productivity)	13	30.6	33.5
My retirement (including the potential to retire and when that may occur)	16	25.8	23.3
**Uncategorized/low endorsed items**			
My spirituality or religiosity	15	26.0	10.4
My housing	19	17.9	5.8
My education or training	20	11.4	31.2

Question stem: How much has each of the following areas of your life been IMPACTED by the COVID-19 pandemic? Response Scale: 1 = *Very Negatively*, 2 = *Somewhat Negatively*, 3 = *Not at all*, 4 = *Somewhat Positively*, 5 = *Positively*, Not Applicable (NA)

^a^Categories (shown in Bold) are a result of Exploratory Factor Analysis (EFA)

^b^Percentages reported out of total sample (N = 520), including those responding NA

#### Protective factors and patient-reported psychological well-being

Mean, range, and SD scores for the CD-RISC, HHI, and PROMIS depression and anxiety are presented in Table 3. For context, we noted that average PROMIS depression (*M* = 52.4) and anxiety (*M* = 56.4) scores in the current sample were higher than comparative pre-pandemic depression (*M* = 48.5) and anxiety (*M* = 49.2) scores in the U.S. cancer population, at a magnitude indicating a clinically meaningful difference, defined as 3 or more points between scores [29]. Further, 22% of patients endorsed depression symptoms and 35% of patients endorsed anxiety symptoms at levels corresponding to moderate-to-severe levels of symptom burden based on established PROMIS benchmarks (mean score > 1 SD).

**Table 3:** Descriptive statistics and correlations between depression, anxiety, COVID-19 impact, and protective factor (N = 520)

	Psychological well-being		COVID-19 impact				Protective Factors	
	PROMIS depression	PROMIS anxiety	Finances	Family	Essential needs	Social	CD-RISC-10	HHI
PROMIS Depression	(.99)							
PROMIS Anxiety	0.76**	(.92)						
Finances	0.24**	0.25**	(.71)					
Family	0.26**	0.31**	0.25**	(.67)				
Essential Needs	0.25**	0.28**	0.25**	0.36**	(.69)			
Social	0.16**	0.15**	0.15**	0.40**	0.28**	(.68)		
CD-RISC-10	− 0.54**	− 0.49**	− 0.15**	− 0.17**	− 0.13*	− 0.08	(.91)	
HHI	− 0.60**	− 0.52**	− 0.15**	− 0.25**	− 0.20**	-0.18**	0.64**	(.89)
Range	41–79.4	40.3–81.6	0–3	0–4	0–3	0–3	5–40	14–48
Mean	52.4	56.4	1.0	1.3	1.6	2.3	27.6	37.2
SD	8.8	9.3	1.1	1.2	1.2	0.9	6.9	5.8

Correlations are unadjusted bivariate Pearson correlations. Entries on the main diagonal are Cronbach’s alphas (*α*) with listwise deletion applied. PROMIS scores are the converted T-scores

*p < .01 **p ≤ .001. HHI Herth Hope Index, CD-RISC-10 Connor-Davidson Resilience Scale

### Inferential analyses

#### Bivariate relationships

Study variables correlated with each other in the predicted directions (see Table 3). As hypothesized, greater COVID-19 impacts were significantly correlated with higher PROMIS depression and anxiety scores, with effect sizes ranging from small to moderate (*r*s = 0.15–0.31). Additionally, hope and resilience protective factors exhibited strong, negative correlations with PROMIS depression and anxiety (rs = − 0.49 to − 0.60), as hypothesized, such that higher HHI and CD-RISC scores were associated with lower PROMIS depression and anxiety.

#### Regression models

All assumptions of multiple regression were tested and met for each model: (1) The relationships between variables are linear; (2) There is no evidence of multicollinearity, as correlations did not exceed 0.8 (see Table 3), VIF scores were well below 10 (Range = 1.01–1.22), and tolerance scores were above 0.2 (Range = 0.82–0.99); (3) The Durbin-Watson statistics show the residuals are independent, as required, with a value close to 2 for each model (Depression = 1.946; Anxiety = 2.043); (4) Plot of standardized residuals versus standardized predicted values indicate that there are no signs of homoscedasticity (i.e., no funneling); (5) P–P plots indicates residuals are normally distributed; and (6) Influential cases/outliers biasing the model, as identified by the Cook’s distance values > 4/n, were not found to alter model outcomes.

As shown in Table 4, each step of the regression models explained a statistically significant amount of variance in depression and anxiety, and the final step was significant for both depression (*R*^2^ = 0.50, *F* = (13, 485) = 36.534, *p* < 0.001) and anxiety (*R*^*2*^ = 0.44, *F* = (13, 487) = 28.837, *p* < *0.0*01). Younger age and woman gender identity were significant predictors of depression and anxiety; lower income was also a predictor of anxiety. With respect to clinical history, general health status was the largest predictor of depression and anxiety, followed by cancer status. The impacts of COVID-19 presented differently across depression and anxiety: financial impact was the sole COVID-19 predictor of depression, while family impact was the sole COVID-19 predictor of anxiety. Neither essential needs nor social impact was a significant predictor of depression or anxiety. With respect to protective factors, both resilience and hope were significant predictors of anxiety and depression, even after controlling for sociodemographic variables, clinical history, and COVID-19 impact. Moreover, resilience and hope carried the largest standardized beta weights and the protective factor block of variables explained the greatest proportion of variance in the overall model (Δ*R*^2^ = 0.22 for depression; Δ*R*^2^ = 0.15 for anxiety) compared to other blocks.

**Table 4:** Hierarchical regression results predicting Depression (Model 1) and Anxiety (Model 2)

Predictors	Model 1: Depression (N = 499)		Model 2: Anxiety (N = 501)	
	B [95% CI]	β	B [95% CI]	β
*Step 1: sociodemographic variables*				
Age	**− 0.10 [− 0.15, − 0.05]**	− 0.13***	**− 0.13 [− 0.19, − 0.08]**	− 0.17***
Gender (1 = Woman)	**1.70 [0.37, 3.02]**	0.08*	**2.64 [1.16, 4.13]**	0.12***
Low income (1 = < 20 k)	1.93 [− 0.26, 4.11]	0.06	**3.39 [0.95, 5.83]**	0.10**
Race (1 = Non-Hispanic White)	− 0.10 [− 1.53, 1.33]	− 0.01	0.16 [− 1.43, 1.75]	− 0.01
**R**^**2**^	**0.05**		**0.08**	
**F**	6.772***		11.426***	
*Step 2: clinical history*				
Health status	**− 1.63 [− 2.31, − 0.94]**	− 0.17***	**− 1.09 [− 1.85, − 0.33]**	− 0.11**
Cancer status (1 = active cancer)	**1.64 [0.34, 2.95]**	0.08*	**1.48 [0.29, 2.94]**	0.07*
Time since diagnosis	0.07 [− 0.01, 0.14]	0.06	0.07 [− 0.02, 0.15]	0.06
**R**^**2**^	**0.22**		**0.20**	
**ΔR**^**2**^	**0.17**		**0.11**	
**ΔF**	34.883***		22.841***	
*Step 3: COVID-19 impact*				
Finances	**0.58 [0, 1.16]**	0.07*	0.61 [− 0.04, 1.26]	0.07
Family	0.41 [− 0.14, 0.96]	0.06	**1.11 [0.49, 1.73]**	0.14***
Essential needs	0.32 [− 0.24, 0.87]	0.04	0.50 [− 0.12, 1.12]	0.06
Social	0.30 [− 0.38, 0.97]	0.03	0.12 [− 0.64, 0.88]	0.01
**R**^**2**^	**0.28**		**0.29**	
**ΔR**^**2**^	**0.06**		**0.09**	
**ΔF**	10.272***		15.489***	
*Step 4: protective factors*				
CD-RISC-10 (resilience)	**− 0.24 [− 0.34, − 0.12]**	− 0.19***	**− 0.25 [− 0.37, − 0.13]**	− 0.18***
HHI (hope)	**− 0.57 [− 0.70, − 0.44]**	− 0.37***	**− 0.46 [− 0.61, − 0.31]**	-0.29***
**R**^**2**^	**0.50**		**0.44**	
**ΔR**^**2**^	**0.22**		**0.15**	
**ΔF**	103.395***		63.977***	
Final model	F(13, 485) = 36.534***		F(13, 487) = 28.837***	

Variables are continuous unless otherwise specified. All regression coefficients are from the final step in the analyses. COVID-19 Impact Well-being category excluded as predictor due to redundancy with outcome variables. Casewise deletion used for missing data. Model statistics at each step and significant predictors shown in bold

*B* unstandardized Beta; *β* standardized Beta

*p ≤ *.*05, **p < *.*01, ***p < *.*001; Sample sizes: Model 1(*N* = 499); Model 2 (*N* = 501)

## Discussion

The current study explored the relationship between COVID-19 impacts in the cancer population and patient-reported depression and anxiety. Findings suggest that people living with cancer experienced a broad range of COVID-19-related impacts in the early phases of the pandemic, especially related to social activity, well-being, and essential needs, and also experienced substantive levels of depression and anxiety symptoms. COVID-19 financial impact was a significant predictor of depression and COVID-19 family impact was a significant predictor of anxiety, even after controlling for sociodemographic and clinical history factors. Additionally, protective factors, hope and resilience, played a significant role in predicting patient-reported depression and anxiety, explaining the largest overall proportion of variance in the models.

Of note, social factors were among the most commonly cited areas impacted by COVID-19 in the current sample, specifically engaging in recreational activities and feeling connected to others and friendships, but were not significant predictors of depression or anxiety. In the context of the pandemic, a disproportionately high number of individuals experienced isolation due to numerous factors including social distancing. The fact that most people endorsed social impact may have resulted in a ceiling effect, such that individuals with both lower and higher levels of depression and anxiety are experiencing and endorsing substantial social isolation. Also of consideration is the fact that social isolation and loneliness have been identified as distinct traits that can exist independently of each other [13, 31] and while isolation might represent the actual lived experience, loneliness is the subjective, negative response to this isolation. The way the current study measured the social impact of COVID-19 might more appropriately capture isolation as opposed to loneliness.

The independent contributions of COVID-19 financial impact on depression and COVID-19 family impact on anxiety, after controlling for covariates, are notable and consistent with pre-pandemic evidence suggesting that cancer patients are already vulnerable to substantial financial burden [32] as well as concerns about the impact of cancer on family members and their well-being [33]. The COVID-19 pandemic has created further disruption to many individuals’ employment and finances [34], as well as placed extraordinary burden on caregivers supporting family members [35].

### Clinical implications

As the cancer population is faced with these many challenges, it is important to consider what existing factors might alleviate the burden of COVID-19. First, systematic multidimensional distress screening may serve a fundamental role in ensuring that cancer patients’ greatest needs across the course of the pandemic are being identified and acted upon through tailored support [36]. Identifying areas of distress and aligning patients with appropriate resources, especially those related to financial and family support services, is always crucial for the cancer community but is even more essential as they continue to navigate the challenges of the pandemic. The current findings suggest that COVID-19 social impact is pervasive, and those who are still practicing social distancing might benefit from using new forms of social engagement (e.g., virtual groups) that can create the space to connect with others when in-person socialization is not possible. Additionally, as we continue to return to in-person activities, organizations should consider how to expand and promote their social events to restore feelings of social connectedness. Second, regression analyses in the current study indicate that both resilience and hope may serve as powerful tools in the cancer experience, even during the COVID-19 pandemic. While causality cannot be asserted in the current study, evidence in the literature suggests that hope and resilience can be enhanced through supportive interventions [37, 38], thus serving as a potentially important pathway towards enhancing patient psychological well-being.

### Study limitations

This study provides important insights into the experiences of the cancer population during the COVID-19 pandemic, but it is not without limitations. While the sample represents a diverse range of cancer diagnoses and clinical characteristics (e.g., time since diagnosis, stage, and status), the sample is predominantly White (83%), women (76%), with some college education (93%). The COVID-19 experiences in historically underserved communities may not be well represented in the current sample, and thus generalizations to those communities should be approached with caution. Further, while the regression findings showing significantly higher depression and anxiety for women is consistent with past research, including research conducting during the pandemic [39], it also points to a need for future work examining the intersection of gender identity with COVID-19 experiences and ensuing mental health, especially when considering how traditional gender roles could affect COVID-19 areas of impact such as finances and family. Additionally, these cross-sectional data were collected at a single timepoint during the first year of the pandemic and, thus, do not account for participants’ mental health prior to their cancer diagnosis or prior to the COVID-19 pandemic. It is possible that participants’ mental health and COVID-19 experiences, including related impact and concerns, shifted over the course of their cancer experiences or over the pandemic timeline as people adjusted to conditions. As previously mentioned, these data come from the baseline survey in a longitudinal study; we will further examine these shifts and within subject changes in mental health over time in subsequent analysis and dissemination efforts. Given the significant relationships seen between certain areas of COVID-19 impact and the patient-reported psychological well-being in the cancer community, one area of interest will be the long-term, and evolving, effect of COVID-19 financial and family impact on depression and anxiety symptom burden. Lastly, only 3% of the sample had tested positive for COVID-19 at the time of the survey, which limited our ability to include this as a predictor in the models, but COVID-19 infection could have a substantial impact on physical and psychological well-being and should be considered in future work.

## Conclusions

COVID-19 continues to threaten the lives and well-being of those living with cancer. While those living with cancer are already at greater risk for experiencing clinical levels of depression and anxiety, COVID-19 could exacerbate the situation. As we continue to recover from the ongoing impact of COVID-19, we must be mindful of the unique experiences and needs of the cancer community. In addition to identifying these needs, we should find actionable ways to support the cancer community, focusing on those risk and protective factors identified in this study as well as others.

## Data Availability

The datasets generated and/or analyzed during the current study are not publicly available due to being part of a larger on-going study but are available from the corresponding author on reasonable request.
